# An Improved ACKF/KF Initial Alignment Method for Odometer-Aided Strapdown Inertial Navigation System

**DOI:** 10.3390/s18113896

**Published:** 2018-11-12

**Authors:** Kang Gao, Shunqing Ren, Guoxing Yi, Jiapeng Zhong, Zhenhuan Wang

**Affiliations:** 1 Space Control and Inertial Technology Research Center, Harbin Institute of Technology, Harbin 150080, China; ygx@hit.edu.cn (G.Y.); zhenhuanwang@gmail.com (Z.W.); 2 HIT (Anshan) Institute of Industrial Technology, Anshan 114000, China; jiapeng.zhong@gmail.com

**Keywords:** strapdown inertial navigation system (SINS), initial alignment, odometer, Sage-Husa adaptive filter, cubature Kalman filter (CKF)

## Abstract

For a land-vehicle strapdown inertial navigation system (SINS), the problem of initial alignment with large misalignment angle in-motion needs to be solved urgently. This paper proposes an improved ACKF/KF initial alignment method for SINS aided by odometer. The SINS error equation with large misalignment angle is established first in the form of an Euler angle. The odometer/gyroscope dead reckoning (DR) error equation is deduced, which makes the observation equation linear when the position is taken as the observation of the Kalman filter. Then, based on the cubature Kalman filter, the Sage-Husa adaptive filter and the characteristics of the observation equation, an improved ACKF/KF method is proposed, which can accomplish initial alignment well in the case of unknown measurement noise. Computer simulation results show that the performance of the proposed ACKF/KF algorithm is superior to EKF, CKF and AEKF method in accuracy and stability, and the vehicle test validates its advantages.

## 1. Introduction

Strapdown inertial navigation systems (SINSs) are widely used in modern navigation applications, especially in the military field because of their advantage of complete autonomy [1,2,3]. Initial alignment plays an important role in SINS, and the accuracy and efficiency of initial alignment affect the SINS capability directly [4,5,6]. The information about speed and location is more available than misalignment. Thus, the main task of initial alignment is to estimate the misalignment [7].

Unlike the alignment on the static base, the alignment on moving base calls for the carrier motion information provided by the external device [8,9]. The GPS is commonly onboard navigation equipment, which can provide high accuracy navigation information [10,11,12]. However, GPS signals are not stable, it can be affected by interference and shielding [13], and the poor stability of GPS is not competent for SINS initial alignment in the military field. With the development of image recognition technology, camera is becoming a promising choice for navigation system [14,15]. While, the visual navigation system calls for the easily identifiable features and known locations on paths, which is not available in the war [16]. Odometers measure the distance increase along the vehicle trajectory. They are a kind of economical, conveniently-deployed and widely-used sensor for land vehicles, and their fully self-contained characteristics are valuable in the military field [17,18], so we choose the odometer to accomplish the in-motion initial alignment.

However, for the SINS initial alignment in-motion with large misalignment angles, the standard Kalman filter is not competent, which leads to the development of non-linear filtering methods in recent years [19,20,21,22,23,24,25,26]. The extended Kalman filter (EKF) is one of the most outstanding nonlinear filters, and in [19] the GPS/SINS tightly coupled integration was completed by state transformation EKF. However, the truncation error caused by one-order Taylor expansion linearization approximation in EKF is large and the calculation of the Jacobian matrix for the alignment model with its high-dimensionality and strong nonlinear character is very complicated, which increases the computational load and brings about unacceptable calculation errors [20]. Based on the idea that approximating a probability distribution is easier than approximating a nonlinear function, Julier et al. proposed the Unscented Kalman Filter (UKF), in which an Unscented Transformation (UT) is used to obtain the statistical properties of the states instead of the local linearization [21]. It is proved that the UKF can achieve two-order accuracy. However, the covariance matrix is sometimes non-positive in high-dimensional systems, and the parameters in the UKF need to be adjusted accordingly [22,23]. In order to solve the high dimensionality nonlinear integral problem, Arasaratnam et al. [24] proposed the spherical radial cubature rule (SRCR), based on which the cubature Kalman filter (CKF) is established., The CKF has been widely used in many applications because of the better numerical stability and accuracy than UKF [25,26].

The Kalman filter should be initialized properly, especially when a coarse estimated initial attitude is asked for, and the statistical characteristics of system noise are needed [27]. However, for a vehicle running freely, the measurement noise changes with the environment and the coarse initial alignment is hard to achieved on a moving-base [28]. Without proper initialization, the Kalman filter- based SINS initial alignment aided by an odometer would hardly converge. Therefore, the adaptive filter method which can estimate the system noise online is necessary. Variance component estimation (VCE) is a kind of adaptive method which can estimate process and measurement noise simultaneously by utilizing residual vectors, but the algorithm is complicated and is rarely used in engineering [29,30,31]. The Sage–Husa adaptive filter is a widely-used adaptive filter, which uses the maximal posterior estimation principle to estimate the system noise Q or measurement noise R to improve the filtering accuracy [7,32,33]. Because of its simplicity and high reliability, the Sage–Husa algorithm has been widely used in engineering, therefore, this algorithm is selected for the estimation of measurement noise to improve the process of SINS initial alignment.

This paper is organized as follows: Section 2 deduces the SINS system equation and the odometer/gyroscope dead reckoning error equation with large misalignment error, and the Kalman filter equation is given. In Section 3, the Cubature Kalman filter and Sage-Husa adaptive filter are introduced, and the improved ACKF/KF method is proposed. The simulation and experiment results and analysis are reported in Section 4. Conclusions are finally drawn in Section 5.

## 2. Nonlinear Initial Alignment Equation 

In order to better understand and deduce the SINS initial alignment aided by odometer, it is necessary to define the related coordinate systems, that is, the inertial frame (i-frame), the Earth frame (e-frame), the navigation frame (n-frame), the vehicle frame (a-frame), the SINS frame (b-frame), the odometer frame (m-frame) and the calculation frame (n’-frame). Here, we denote the geographic coordinate system “east-north-up (ENU)” as the n-frame and select the “right-front-up (RFU)” coordinate system as the a-frame. As the odometer is installed on the front wheel of the vehicle and measures the front speed of the vehicle, thus the m-frame is coincident with the a-frame. There is a small misaligned angle between the b-frame and the a-frame because of the installation error.

### 2.1. SINS Error Equation with Large Misalignment Angle

In general, there are misalignment angles between the n-frame and n′-frame. Denote the transformation from n-frame to n′-frame by rotating around *z*-axis, *x*-axis, and *y*-axis with $\alpha_{z}$,$\alpha_{x}$,$\alpha_{y}$, and the Euler angle is defined as $\alpha ≜\left[\alpha_{z}，\alpha_{x}，\alpha_{y}\right]$.

In practical applications, horizontal alignment is easier to achieve by mechanical means, such as designing the SINS installation surface to be a parallel structure to ensure that the horizontal misalignment angle is small. However, the azimuth angle is more complex. Owing to this, we can assume that the horizontal misalignment angles are small angles, only the azimuth misalignment angle is considered to be large one. Then the attitude transformation matrix from n-frame to n′-frame can be simplified as:

(1) $$C_{n}^{n^{\prime }}=C_{\alpha_{y}}C_{\alpha_{x}}C_{\alpha_{z}}\approx \left[\begin{array}{ccc} \cos\alpha_{z} & \sin\alpha_{z} & -\alpha_{y}\\ -\sin\alpha_{z} & \cos\alpha_{z} & \alpha_{x}\\ \alpha_{y}\cos\alpha_{z}+\alpha_{x}\sin\alpha_{z} & \alpha_{y}\sin\alpha_{z}-\alpha_{x}\cos\alpha_{z} & 1 \end{array}\right]\;$$

Denote the angular velocity of n′-frame with respect to n-frame is $\omega_{nn^{\prime }}^{n^{\prime }}$

(2) $$\omega_{nn^{\prime }}^{n^{\prime }}=C_{\alpha_{y}}C_{\alpha_{x}}\left[\begin{array}{c} 0\\ 0\\ {\dot{\alpha }}_{z} \end{array}\right]+C_{\alpha_{y}}\left[\begin{array}{c} {\dot{\alpha }}_{x}\\ 0\\ 0 \end{array}\right]+\left[\begin{array}{c} 0\\ {\dot{\alpha }}_{y}\\ 0 \end{array}\right]=C_{\omega }\dot{\alpha }\;$$

The differential equation of Euler angle is obtained as:

(3) $$\dot{\alpha }=C_{\omega }^{-1}\omega_{nn^{\prime }}^{n^{\prime }}\;$$

where:

(4) $$C_{\omega }^{-1}=\left[\begin{array}{ccc} 1 & 0 & \alpha_{y}\\ 0 & 1 & -\alpha_{x}\\ -\alpha_{y} & 0 & 1 \end{array}\right]\;$$

The matrix differential equation of SINS is

(5) $${\dot{C}}_{b}^{n}=C_{b}^{n}\left[\omega_{ib}^{b}\times \right]-\left[\omega_{in}^{n}\times \right]C_{b}^{n}\;$$

where, $C_{b}^{n}$ denotes the attitude transformation matrix from b-frame to n-frame, $\omega_{ib}^{b}$ is the body angular rate measured by gyroscopes denoted in b-frame, $\omega_{in}^{n}$ is the angular rate of n-frame with respect to i-frame denoted in n-frame.

The attitude matrix differential equation including the calculation error is:

(6) $${\dot{C}}_{b}^{n^{\prime }}=C_{b}^{n^{\prime }}\left[{\tilde{\omega }}_{ib}^{b}\times \right]-\left[\omega_{in^{\prime }}^{n^{\prime }}\times \right]C_{b}^{n^{\prime }}\;$$

where, ${\tilde{\omega }}_{ib}^{b}=\omega_{ib}^{b}+\varepsilon^{b}$, $\omega_{in^{\prime }}^{n^{\prime }}=\omega_{in}^{n}+\delta \omega_{in^{\prime }}^{n^{\prime }}$, $\varepsilon^{b}$ is gyroscope bias.

The attitude error matrix is defined as:

(7) $$C_{b}^{n}=C_{n^{\prime }}^{n}C_{b}^{n^{\prime }}=C_{b}^{n^{\prime }}+\delta C_{b}^{n^{\prime }}\;$$

Namely:

(8) $$\delta C_{b}^{n^{\prime }}=C_{b}^{n}-C_{b}^{n^{\prime }}\;$$

(9) $$\delta C_{b}^{n^{\prime }}=\left(C_{n^{\prime }}^{n}-I\right)C_{b}^{n^{\prime }}\;$$

The two sides of Equations (8) and (9) are differentiated respectively, subtracted and arranged as follows:

(10) $${\dot{C}}_{n^{\prime }}^{n}+C_{n^{\prime }}^{n}\left[\varepsilon^{n^{\prime }}\times \right]-C_{n^{\prime }}^{n}\left[\omega_{in^{\prime }}^{n^{\prime }}\times \right]+\left[\omega_{in^{\prime }}^{n^{\prime }}\times \right]C_{n^{\prime }}^{n}+\left[\delta \omega_{in^{\prime }}^{n^{\prime }}\times \right]C_{n^{\prime }}^{n}=0\;$$

Substituting ${\dot{C}}_{n^{\prime }}^{n}=C_{n^{\prime }}^{n}\left[\omega_{nn^{\prime }}^{n^{\prime }}\times \right]$ into Equation (10):

(11) $$\omega_{nn^{\prime }}^{n^{\prime }}+\varepsilon^{n^{\prime }}-\omega_{in^{\prime }}^{n^{\prime }}+C_{n}^{n^{\prime }}\left(\omega_{in^{\prime }}^{n^{\prime }}+\delta \omega_{in^{\prime }}^{n^{\prime }}\right)=0\;$$

By substituting Equation (2) into Equation (11), the nonlinear attitude error equation of SINS represented by Euler angle can be obtained:

(12) $$\dot{\alpha }=C_{\omega }^{-1}\left(-\varepsilon^{n^{\prime }}+\left(I-C_{n}^{n^{\prime }}\right)\omega_{in^{\prime }}^{n^{\prime }}-C_{n}^{n^{\prime }}\delta \omega_{in^{\prime }}^{n^{\prime }}\right)\;$$

According to the SINS specific force equation, when the error is not considered, the ideal velocity equation is:

(13) $${\dot{v}}^{n}=C_{b}^{n}f^{b}-\left(2\omega_{ie}^{n}+\omega_{en}^{n}\right)\times v^{n}+g^{n}\;$$

where, $v^{n}$ is the velocity relative to n-frame measured by SINS, $f^{b}$ is the specific force measured by accelerometers in b-frame, $\omega_{en}^{n}$ is the angular rate of the e-frame with respect to n-frame, and $g^{n}$ is the gravity vector.

The speed equation including the calculation error is:

(14) $${\dot{v}}^{n^{\prime }}=C_{b}^{n^{\prime }}{\tilde{f}}^{b}-\left(2\omega_{ie}^{n^{\prime }}+\omega_{en^{\prime }}^{n^{\prime }}\right)\times v^{n^{\prime }}+g^{n^{\prime }}\;$$

where, $v^{n}=v^{n^{\prime }}+\delta v^{n^{\prime }}$, $\omega_{ie}^{n}=\omega_{ie}^{n^{\prime }}+\delta \omega_{ie}^{n^{\prime }}$, $\omega_{en}^{n}=\omega_{en^{\prime }}^{n^{\prime }}+\delta \omega_{en^{\prime }}^{n^{\prime }}$, $g^{n}=g^{n^{\prime }}+\delta g^{n^{\prime }}$, ${\tilde{f}}^{b}=f^{b}+\nabla^{b}$, $C_{b}^{n}=C_{n^{\prime }}^{n}C_{b}^{n^{\prime }}=C_{b}^{n^{\prime }}+\delta C_{b}^{n^{\prime }}$.

Equation (13) minus Equation (14):

(15) $$\begin{array}{l} \delta {\dot{v}}^{n^{\prime }}=\left(C_{n^{\prime }}^{n}-I\right)C_{b}^{n^{\prime }}{\tilde{f}}^{b}-C_{n^{\prime }}^{n}C_{b}^{n^{\prime }}\nabla^{b}-\left(2\omega_{ie}^{n^{\prime }}+\omega_{en^{\prime }}^{n^{\prime }}\right)\times \delta v^{n^{\prime }}\\ \;-\left(2\delta \omega_{ie}^{n^{\prime }}+\delta \omega_{en^{\prime }}^{n^{\prime }}\right)\times v^{n^{\prime }}-\left(2\delta \omega_{ie}^{n^{\prime }}+\delta \omega_{en^{\prime }}^{n^{\prime }}\right)\times \delta v^{n^{\prime }}+\delta g^{n^{\prime }} \end{array}$$

where,$C_{n^{\prime }}^{n}$ is defined by Equation (1). The above Equation (15) is the speed error equation of SINS with large misalignment angle.

The position error equation of SINS defined as:

(16) $$\begin{array}{l} \delta \dot{L}=\hat{\dot{L}}-\dot{L}=\frac{1}{R_{M}+h}\delta v_{N}^{n}\\ \delta \dot{\lambda }=\hat{\dot{\lambda }}-\dot{\lambda }=\frac{\sec L}{R_{N}+h}\delta v_{E}^{n}+\frac{v_{E}^{n}\sec L\tan L}{R_{N}+h}\delta L \end{array}$$

where, L, λ and h denote the longitude, latitude and altitude respectively. $R_{M}$ and $R_{N}$ denote the radius of curvature in meridian and in prime vertical, respectively.

### 2.2. Odometer/Gyroscopes Dead Reckoning Error Equation

The dead reckoning (DR) is a commonly used carrier independent positioning technique that uses posture, heading, and mileage information to calculate the vehicle’s position relative to the starting point. In this paper, the angular rate measured by gyroscopes of the IMU and the velocity measured by odometer are used to realize the dead reckoning. The dead reckoning algorithm includes the position updating algorithm and the attitude updating algorithm.

Define the b-frame with respect to the n-frame is $C_{bD}^{n}$, which is calculated by the gyroscopes of IMU, and the subscript *D* means the variable output by the dead reckoning algorithm.

The velocity measured by odometer expressed in n-frame is:

(17) $$v_{D}^{n}=C_{b}^{n}v_{D}^{b}\;$$

The position differential equations are similar to the SINS position update equations and can be expressed as:

(18) $$\left\{ \begin{array}{l} {\dot{L}}_{D}=\frac{v_{DN}^{n}}{R_{M}+h_{D}}\\ {\dot{\lambda }}_{D}=\frac{v_{DE}^{n}\sec L_{D}}{R_{N}+h_{D}} \end{array}\right.\;$$

where, $v_{D}^{n}={\left[\begin{array}{ccc} v_{DE}^{n} & v_{DN}^{n} & v_{DU}^{n} \end{array}\right]}^{T}$, $p_{D}={\left[\begin{array}{ccc} L_{D} & \lambda_{D} & h_{D} \end{array}\right]}^{T}$.

Then, considering the velocity error, the DR horizontal position error can be expressed as:

(19) $$\left\{ \begin{array}{l} \delta {\dot{L}}_{D}={\hat{\dot{L}}}_{D}-{\dot{L}}_{D}=\frac{1}{R_{M}+h_{D}}\delta v_{DN}^{n}\\ \delta {\dot{\lambda }}_{D}={\hat{\dot{\lambda }}}_{D}-{\dot{\lambda }}_{D}=\frac{\sec L_{D}}{R_{N}+h_{D}}\delta v_{DE}^{n}+\frac{v_{DE}^{n}\sec L_{D}\tan L_{D}}{R_{N}+h_{D}}\delta L_{D} \end{array}\right.\;$$

We denote the IMU installation error angle as $\theta ={\left[\begin{array}{ccc} \theta_{x} & \theta_{y} & \theta_{z} \end{array}\right]}^{T}$, that is the misalignment angle between b-frame and m-frame. Assuming the IMU installation error angle is small and taking the odometer scale factor error $\delta K_{D}$ and attitude error into account, the speed measured by odometer $v_{D}$ expressed in n-frame is:

(20) $${\hat{v}}_{D}^{n}=C_{n}^{n^{\prime }}C_{b}^{n}\left[\begin{array}{c} -\theta_{z}\\ 1+\delta K_{D}\\ \theta_{x} \end{array}\right]v_{D}\;$$

The velocity error is defined as the calculated value minus the true value, as:

(21) $$\delta v_{D}^{n}={\hat{v}}_{D}^{n}-v_{D}^{n}\;$$

Only taking the horizontal velocity into account, the component form of velocity error is:

(22) $$\left\{ \begin{array}{l} \delta v_{DE}^{n}=[\theta_{x}\left(T_{13}\cos\alpha_{z}+T_{23}\sin\alpha_{z}-T_{33}\alpha_{y}\right)-\theta_{z}\left(T_{11}\cos\alpha_{z}+T_{21}\sin\alpha_{z}-T_{31}\alpha_{y}\right)\\ \;+\left(1+\delta K_{D}\right)\left(T_{12}\cos\alpha_{z}+T_{22}\sin\alpha_{z}-T_{32}\alpha_{y}\right)-T_{12}]v_{D}\\ \delta v_{DN}^{n}=[\theta_{x}\left(-T_{13}\sin\alpha_{z}+T_{23}\cos\alpha_{z}+T_{33}\alpha_{x}\right)-\theta_{z}\left(-T_{11}\sin\alpha_{z}+T_{21}\cos\alpha_{z}+T_{31}\alpha_{x}\right)\\ \;+\left(1+\delta K_{D}\right)\left(-T_{12}\sin\alpha_{z}+T_{22}\cos\alpha_{z}+T_{32}\alpha_{x}\right)-T_{22}]v_{D} \end{array}\right.\;$$

where, $\delta v_{DE}^{n}$ and $\delta v_{DN}^{n}$ are the east and north velocity error, $T_{ij}$ is the element of $C_{b}^{n}$.

By substituting Equation (22) into Equation (19), the calculated position error equation can be obtained:

(23) $$\left\{ \begin{array}{l} \delta {\dot{L}}_{D}=\frac{1}{R_{M}+h_{D}}[\theta_{x}\left(-T_{13}\sin\alpha_{z}+T_{23}\cos\alpha_{z}+T_{33}\alpha_{x}\right)-\theta_{z}\left(-T_{11}\sin\alpha_{z}+T_{21}\cos\alpha_{z}+T_{31}\alpha_{x}\right)\\ \;+\left(1+\delta K_{D}\right)\left(-T_{12}\sin\alpha_{z}+T_{22}\cos\alpha_{z}+T_{32}\alpha_{x}\right)-T_{22}]v_{D}\\ \delta {\dot{\lambda }}_{D}=\frac{\sec L_{D}}{R_{N}+h_{D}}[\theta_{x}\left(T_{13}\cos\alpha_{z}+T_{23}\sin\alpha_{z}-T_{33}\alpha_{y}\right)-\theta_{z}\left(T_{11}\cos\alpha_{z}+T_{21}\sin\alpha_{z}-T_{31}\alpha_{y}\right)\\ \;+\left(1+\delta K_{D}\right)\left(T_{12}\cos\alpha_{z}+T_{22}\sin\alpha_{z}-T_{32}\alpha_{y}\right)-T_{12}]v_{D}+\frac{v_{DE}^{n}\sec L_{D}\tan L_{D}}{R_{N}+h_{D}}\delta L_{D} \end{array}\right.\;$$

### 2.3. Kalman Filter Equation

Taking attitude errors, horizontal velocity errors, SINS positioning errors, DR positioning errors, constant gyro drifts, accelerometer biases, misalignment angles and odometer factor error into account, the state error equation can be expressed as:

(24) $$\dot{x}=f\left(x\right)+w\;$$

where, the state vector is:

(25) $$\begin{array}{l} x=[\left[\begin{array}{ccc} \alpha_{x} & \alpha_{y} & \alpha_{z} \end{array}\right]\;\left[\begin{array}{cc} \delta v_{E}^{n} & \delta v_{N}^{n} \end{array}\right]\;\left[\begin{array}{cc} \delta L & \delta \lambda \end{array}\right]\;\left[\begin{array}{cc} \delta L_{D} & \delta \lambda_{D} \end{array}\right]\\ {\;\left[\begin{array}{ccc} \varepsilon_{x} & \varepsilon_{y} & \varepsilon_{z} \end{array}\right]\;\left[\begin{array}{ccc} \nabla_{x} & \nabla_{y} & \nabla_{z} \end{array}\right]\;\left[\begin{array}{ccc} \theta_{x} & \theta_{z} & \delta K_{D} \end{array}\right]]}^{T} \end{array}$$

The position difference between SINS and DR is used as the observation:

(26) $$z=\left[\begin{array}{c} L-L_{D}\\ \lambda -\lambda_{D} \end{array}\right]=\left[\begin{array}{c} \delta L-\delta L_{D}\\ \delta \lambda -\delta \lambda_{D} \end{array}\right]\;$$

And the observing matrix ***H*** can be indicated as:

(27) $$H=\left[\begin{array}{ccccccc} 0_{2\times 3} & 0_{2\times 2} & I_{2\times 2} & -I_{2\times 2} & 0_{2\times 3} & 0_{2\times 3} & 0_{2\times 3} \end{array}\right]\;$$

The advantage of deriving the DR error equation is that the linear measurement equation can be obtained when the position error is taken as the observation of Kalman filter as shown in Equation (27). Owing to this, the calculation process of the initial alignment can be sharply reduced. If the odometer output expression in b-frame ${\hat{v}}_{D}^{b}$ is taken as the observation directly, the measurement equation will be very complicated with strong nonlinear. 

## 3. Improved Adaptive CKF/KF Method 

### 3.1. Cubature Kalman Filter

Cubature Kalman Filter (CKF) is a nonlinear filtering algorithm proposed by Ienkaran Arasaratnam and Simon Haykin to solve the problem of multi-dimensional state estimation. The core of CKF is the spherical-radial cubature rule, which is used to solve the multi-dimensional integral problem in nonlinear Bayesian filtering [24].

The state space form of discrete nonlinear system is described as:

(28) $$\left\{ \begin{array}{l} x_{k}=f\left(x_{k-1}\right)+w_{k-1}\\ z_{k}=h\left(x_{k}\right)+v_{k} \end{array}\right.\;$$

where, $x_{k}$ and $z_{k}$ are the state vector and the measurement vector respectively, $f\left(x_{k-1}\right)$ is nonlinear state function, $h\left(x_{k}\right)$ is nonlinear measurement vector function, $w_{k}∼N\left(0,Q_{k}\right)$ is the random system noise, $v_{k}∼N\left(0,R_{k}\right)$ is the random measurement noise.

According to the extended three-dimension spherical-radial rule, the calculation of a standard Gaussian weighted integral is:

(29) $$I_{N}\left(g\right)=\int_{R}g\left(x\right)N\left(x;0,I\right)dx\approx \sum_{i=1}^{m}\omega_{i}g\left(\xi_{i}\right)\;$$

where, g(·) is arbitrary nonlinear function, and the cubature points are defined as follows:

(30) $$\left\{ \begin{array}{l} \xi_{i}=\sqrt{\frac{m}{2}}{\left[1\right]}_{i}\\ \omega_{i}=\frac{1}{m},\;i=1,\ldots m,m=2n \end{array}\right.\;$$

The CKF filtering algorithm using the cubature points $\left[\xi_{i},\omega_{i}\right]$ is as follows

▪ Time update:
  (1) Assume that the posterior density function $p\left(x_{k-1}\right)=N\left({\hat{x}}_{k-1|k-1},P_{k-1|k-1}\right)$ is known, the Cholesky Decomposition of error covariance $P_{k-1|k-1}$ is:
    (31) $$P_{k-1|k-1}=S_{k-1|k-1}S_{k-1|k-1}^{T}\;$$
  (2) Calculate the cubature points:
    (32) $$X_{i,k-1|k-1}=S_{k-1|k-1}\xi_{i}+{\hat{x}}_{k-1|k-1}\;$$
  (3) Propagate cubature points through the state equation:
    (33) $$X_{i,k|k-1}^{*}=f\left(X_{i,k-1|k-1}\right)\;$$
  (4) Estimate state predictions:
    (34) $${\hat{x}}_{k|k-1}=\frac{1}{m}\sum_{i=1}^{m}X_{i,k|k-1}^{*}\;$$
  (5) Estimate the state error covariance predictor:
    (35) $$P_{k|k-1}=\frac{1}{m}\sum_{i=1}^{m}X_{i,k|k-1}^{*}X_{i,k|k-1}^{*\;T}-{\hat{x}}_{k|k-1}{\hat{x}}_{k|k-1}^{T}+Q_{k-1}\;$$
▪ Measurement update:
  (1) Cholesky decomposition of $P_{k|k-1}$:
    (36) $$P_{k|k-1}=S_{k|k-1}S_{k|k-1}^{T}\;$$
  (2) Calculate cubature points:
    (37) $$X_{i,k|k-1}=S_{k|k-1}\xi_{i}+{\hat{x}}_{k|k-1}\;$$
  (3) Propagate cubature points by the measurement equation:
    (38) $$Z_{i,k|k-1}=h\left(X_{i,k|k-1}\right)\;$$
  (4) Measurement prediction:
    (39) $${\hat{z}}_{k|k-1}=\frac{1}{m}\sum_{i=1}^{m}Z_{i,k|k-1}\;$$
  (5) Estimate the self-correlation covariance matrix:
    (40) $$P_{zz,k|k-1}=\frac{1}{m}\sum_{i=1}^{m}Z_{i,k|k-1}Z_{i,k|k-1}^{T}-{\hat{z}}_{k|k-1}{\hat{z}}_{k|k-1}^{T}+R_{k}\;$$
  (6) Estimate the mutual correlation covariance matrix:
    (41) $$P_{xz,k|k-1}=\sum_{i=1}^{m}\omega_{i}X_{i,k|k-1}Z_{i,k|k-1}^{T}-{\hat{x}}_{k|k-1}{\hat{z}}_{k|k-1}^{T}\;$$
  (7) Estimate the gain matrix:
    (42) $$K_{k}=P_{xz,k|k-1}P_{zz,k|k-1}^{-1}\;$$
  (8) Calculate the state estimation:
    (43) $${\hat{x}}_{k|k}={\hat{x}}_{k|k-1}+K_{k}\left(z_{k}-{\hat{z}}_{k|k-1}\right)\;$$
  (9) Calculate the state error covariance estimation:
    (44) $$P_{k|k}=P_{k|k-1}-K_{k}P_{zz,k|k-1}K_{k}^{T}\;$$

As can be seen, CKF calculates a set of points with an even number of equal weights according to the spherical-radial cubature rule, which captures the mean and variance of the Gaussian distribution variables completely, and after the transformation of the nonlinear system equation, the precision can reach third-order or higher. So it is not necessary to linearize the nonlinear model, and it is independent of the non-linear equations of the practical system model.

### 3.2. Sage-Husa Adaptive Filter

In theory, the optimal estimation of the state can be obtained only if the structural parameters and the statistical parameters of the stochastic dynamic system are accurately known. However, in practical application, the above two kinds of parameters are more or less inaccurate, resulting in the accuracy of Kalman filter is reduced, the serious can also cause filtering divergence. Sage and Husa proposed an adaptive filtering algorithm which can estimate the noise parameters of the system in real time by measuring the output, but it is often impossible to estimate all the noise parameters (System noise mean and variance, measurement noise mean and variance). Therefore, only the most commonly used and more effective adaptive algorithm for measurement noise variance matrix is given.

The system is assumed to be linear and described as:

(45) $$\left\{ \begin{array}{l} x_{k}=\Phi_{k/k-1}x_{k-1}+\Gamma_{k-1}w_{k-1}\\ z_{k}=H_{k}x_{k}+v_{k} \end{array}\right.\;$$

There are several conditions that need to be fulfilled by $w_{k}$ and $v_{k}$:

(46) $$\left\{ \begin{array}{ll} E\left[w_{k}\right]=0, & E\left[w_{k}w_{j}^{T}\right]=Q_{k}\delta_{kj}\\ E\left[v_{k}\right]=0, & E\left[v_{k}v_{j}^{T}\right]=R_{k}\delta_{kj}\\ E\left[w_{k}v_{j}^{T}\right]=0 \end{array}\right.\;$$

where, the variance matrix of the measurement noise $R_{k}$ is assumed to be unknown and the adaptive estimation method of $R_{k}$ is shown as follows.

According to the steps of the Kalman filter, the estimated errors which are also called innovations can be calculated:

(47) $$\begin{array}{l} \varepsilon_{k}=z_{k}-{\hat{z}}_{k|k-1}\\ \;=H_{k}x_{k}+v_{k}-H_{k}{\hat{x}}_{k|k-1}\\ \;=H_{k}{\tilde{x}}_{k|k-1}+v_{k} \end{array}$$

In consideration of the one step estimated error of state ${\tilde{x}}_{k/k-1}$ and measurement noise $v_{k}$ are both zero mean value, we can demonstrate that the mean value of innovation is zero. Furthermore, ${\tilde{x}}_{k/k-1}$ and $v_{k}$ are uncorrelated. The following equation can be developed by taking the variances on both sides of Equation (47):

(48) $$E\left[\varepsilon_{k}\varepsilon_{k}^{T}\right]=H_{k}P_{k/k-1}H_{k}^{T}+R_{k}\;$$

Rewriting Equation (48), the variance matrix of the measurement noise $R_{k}$ can be calculated as follows:

(49) $$R_{k}=E\left[\varepsilon_{k}\varepsilon_{k}^{T}\right]-H_{k}P_{k/k-1}H_{k}^{T}\;$$

where, $E\left[\varepsilon_{k}\varepsilon_{k}^{T}\right]$ stands for the lumped mean value of random sequence in theory. Nevertheless, it ought to be replaced by time averaged value in adaptive filter method. $R_{k}$ can be established by equal linear weighting recursive estimation method as follows:

(50) $$\begin{array}{l} {\hat{R}}_{k}=\frac{1}{k}\sum_{i=1}^{k}\left(\varepsilon_{i}\varepsilon_{i}^{T}-H_{i}P_{i|i-1}H_{i}^{T}\right)\\ \;=\frac{1}{k}\left[\sum_{i=1}^{k-1}\left(\varepsilon_{i}\varepsilon_{i}^{T}-H_{i}P_{i|i-1}H_{i}^{T}\right)+(\varepsilon_{k}\varepsilon_{k}^{T}-H_{k}P_{k|k-1}H_{k}^{T})\right]\\ \;=\frac{1}{k}\left[\left(k-1){\hat{R}}_{k-1}+(\varepsilon_{k}\varepsilon_{k}^{T}-H_{k}P_{k|k-1}H_{k}^{T}\right)\right]\\ \;=(1-\frac{1}{k}){\hat{R}}_{k-1}+\frac{1}{k}(\varepsilon_{k}\varepsilon_{k}^{T}-H_{k}P_{k|k-1}H_{k}^{T}) \end{array}$$

Besides the equal linear weighting method, in order to decrease the impact of obsolete measurement noise, Equation (50) can be rewritten with fading memory exponent weighted average method as follows:

(51) $${\hat{R}}_{k}=\left(1-\beta_{k}\right){\hat{R}}_{k-1}+\beta_{k}\left(\varepsilon_{k}\varepsilon_{k}^{T}-H_{k}P_{k|k-1}H_{k}^{T}\right)\;$$

(52) $$\beta_{k}=\frac{\beta_{k-1}}{\beta_{k-1}+b}\;$$

where initial value $\beta_{0}=1$ and 0<b<1 denotes the fading factor. When *k* is enough large, $\beta_{k}\approx 1-b$ can be obtained within the approximation. The smaller the fading factor *b* is, the less the influence of obsolete noise. Normally, b=0.9~0.999.

As the estimation of $R_{k}$ in adaptive filter has an effect on the gain calculation, the filter calculation loop of the adaptive filter is no longer simple and linear as the standard Kalman filter, leading the adaptive filter to be an essentially unusually complex nonlinear system. In theory, it is very difficult to analyze the observability and stability of adaptive filter, so the number of adaptive parameters should be minimized in practical use, which is helpful to ensure the effectiveness of filter.

### 3.3. ACKF/KF Method

The Sage-Husa adaptive Kalman filter has the same structure as the standard Kalman filter, but the measurement noise covariance is estimated online. The structure of CKF also maintains the standard Kalman filter structure, only adopts the cubature rule to update the calculation of error covariance for the nonlinear system. Both of the Sage-Husa adaptive Kalman filter and CKF are extensions of the standard Kalman filter, which makes it possible to apply Sage-Husa adaptive strategy to CKF, namely the adaptive cubature Kalman filtering (ACKF).

When the difference between the position of DR and SINS is used as the observation, the linear measurement equation can be obtained. Therefore, the ACKF algorithm can be simplified, that is, the time update of the state and the covariance of the state estimation is carried out by the ACKF method for the state equation. For the observation equation, the standard KF is used for measurement update, that is, the ACKF/KF algorithm. This can greatly reduce the calculation of the initial alignment and ensure the accuracy.

In the case of nonlinear state equation, linear measurement equation with unknown measurement noise, ACKF/KF algorithm is as follows:

▪ The system description:
  (53) $$\left\{ \begin{array}{l} x_{k}=f\left(x_{k-1}\right)+w_{k-1}\\ z_{k}=H_{k}x_{k}+v_{k} \end{array}\right.\;$$
  (54) $$\left\{ \begin{array}{ll} E[w_{k}]=0, & E[w_{k}w_{j}^{T}]=Q_{k}\delta_{kj}\\ E[v_{k}]=0, & E[v_{k}v_{j}^{T}]=R_{k}\delta_{kj}\\ E[w_{k}v_{j}^{T}]=0 \end{array}\right.\;$$
  where, $R_{k}$ is unknown.
▪ Time update:
  (1) Assume that the posterior density function $p\left(x_{k-1}\right)=N\left({\hat{x}}_{k-1|k-1},P_{k-1|k-1}\right)$ is known, the Cholesky Decomposition of error covariance $P_{k-1|k-1}$ is:
    (55) $$P_{k-1|k-1}=S_{k-1|k-1}S_{k-1|k-1}^{T}\;$$
  (2) Calculate the cubature points:
    (56) $$X_{i,k-1|k-1}=S_{k-1|k-1}\xi_{i}+{\hat{x}}_{k-1|k-1}\;$$
  (3) Propagate cubature points through the state equation:
    (57) $$X_{i,k|k-1}^{*}=f\left(X_{i,k-1|k-1}\right)\;$$
  (4) Estimate state predictions:
    (58) $${\hat{x}}_{k|k-1}=\frac{1}{m}\sum_{i=1}^{m}X_{i,k|k-1}^{*}\;$$
  (5) Estimate the state error covariance predictor:
    (59) $$P_{k|k-1}=\frac{1}{m}\sum_{i=1}^{m}X_{i,k|k-1}^{*}X_{i,k|k-1}^{*\;T}-{\hat{x}}_{k|k-1}{\hat{x}}_{k|k-1}^{T}+Q_{k-1}\;$$
▪ Measurement update
  (1) Predict the measurement:
    (60) $${\hat{z}}_{k|k-1}=H_{k}{\hat{x}}_{k|k-1}\;$$
  (2) Calculate the innovation:
    (61) $$\varepsilon_{k}=z_{k}-{\hat{z}}_{k|k-1}\;$$
  (3) Estimate the measurement noise:
    (62) $${\hat{R}}_{k}=\left(1-\beta_{k}\right){\hat{R}}_{k-1}+\beta_{k}\left(\varepsilon_{k}\varepsilon_{k}^{T}-H_{k}P_{k|k-1}H_{k}^{T}\right)\;$$
  (4) Estimate the self-correlation covariance matrix:
    (63) $$P_{zz,k|k-1}=H_{k}P_{k|k-1}H_{k}^{T}+{\hat{R}}_{k}\;$$
  (5) Estimate the mutual correlation covariance matrix:
    (64) $$P_{xz,k|k-1}=P_{k|k-1}H_{k}^{T}\;$$
  (6) Estimate the gain matrix:
    (65) $$K_{k}=P_{xz,k|k-1}P_{zz,k|k-1}^{-1}\;$$
  (7) Calculate the state estimation:
    (66) $${\hat{x}}_{k|k}={\hat{x}}_{k|k-1}+K_{k}\left(z_{k}-{\hat{z}}_{k|k-1}\right)\;$$
  (8) Calculate the state error covariance estimation:
    (67) $$P_{k|k}=P_{k|k-1}-K_{k}P_{zz,k|k-1}K_{k}^{T}\;$$

According to the structural features of Sage-Husa adaptive filtering and cubature Kalman filtering and the characteristics of system equations, the adaptive filtering and cubature Kalman filtering are effectively combined and simplified, and an improved ACKF/KF algorithm is proposed. The introduction of adaptive adjustment strategy in CKF can not only guarantee the filtering accuracy of the nonlinear system, but also make the filtering immune to the change of measurement noise. The combination of the advantages of the two technologies further improves the accuracy and stability of the filtering algorithm, which has important engineering application value.

## 4. Simulation and Experiment

To verify the performance of the proposed ACKF/KF algorithm, simulations and experiments are performed in this section.

### 4.1. Simulation and Analysis

**Figure 1:**
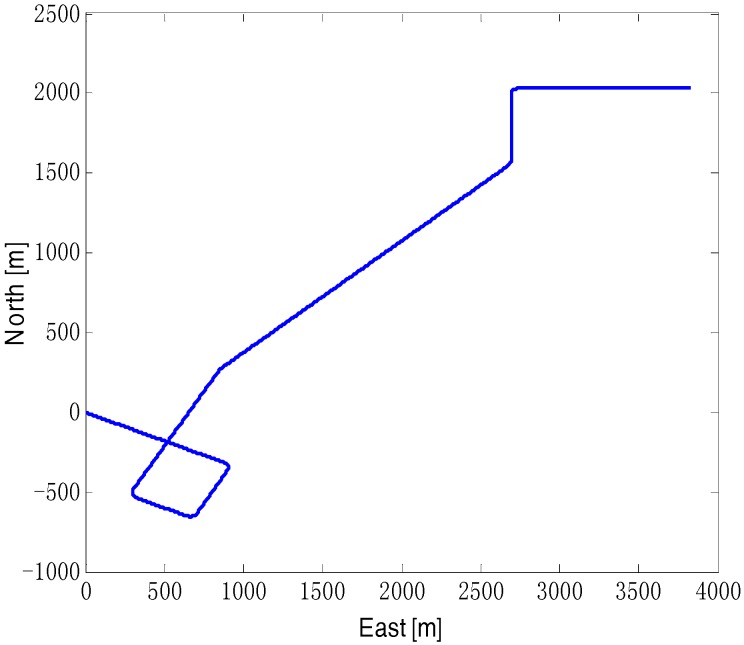
The vehicle’s simulation trajectory.

The design of the vehicle motion trajectory as shown in Figure 1, the vehicle maneuvering mode including constant speed, acceleration, yaw and pitch, the simulation time is 900 s, distance is 5.3 km. The initial value of the attitude angle is [0° 0° −117°], the position initial value is longitude 126.6°, Latitude 45.7°. The standard deviation of position random white noise at the beginning is 10 m of positional error, and the standard deviation of position random white noise increases to 30 m during 400 s to 500 s, simulating a section of road with poor traffic.

The SINS is comprised of a triad of orthogonal gyroscopes with drift of 0.05°/h and noise of 0.01°/(h/$\sqrt{\mathrm{Hz}}$), and a triad of orthogonal accelerometers with bias of 500 µg and noise of 100 µg/$\sqrt{\mathrm{Hz}}$, and the IMU sampling rate is 100 Hz. The odometer scale factor error is 0.002 and the noise of velocity is 0.02 m/s.

Extended Kalman Filter (EKF), Adaptive Extended Kalman filter (AEKF), Cubature Kalman filter (CKF) as the comparisons of the ACKF/KF are set up. The attitude misalignment angle of the simulation is [1° 1° 15°] and the filter parameters are set as follows:

Filter initial value:

$$x_{0}=0_{18\times 1}.$$

Initial estimate error covariance:

$$\begin{array}{l} P_{0}=\mathrm{diag}[\left[\begin{array}{ccc} 5{\;}^{\circ } & 5{\;}^{\circ } & 30{\;}^{\circ } \end{array}\right]\;\left[\begin{array}{cc} 1\;m/s & 1\;m/s \end{array}\right]\;\left[\begin{array}{cc} 50\;m & 50\;m \end{array}\right]\;\left[\begin{array}{cc} 50\;m & 50\;m \end{array}\right]\\ {\;\left[\begin{array}{ccc} \varepsilon_{x} & \varepsilon_{y} & \varepsilon_{z} \end{array}\right]\;\left[\begin{array}{ccc} \nabla_{x} & \nabla_{y} & \nabla_{z} \end{array}\right]\;\left[\begin{array}{ccc} 5{\;}^{\circ } & 5{\;}^{\circ } & 0.01 \end{array}\right]]}^{2} \end{array}$$

Process noise Covariance:

$$Q=\mathrm{diag}{\left[\begin{array}{cccccccccc} \varepsilon & \varepsilon & \varepsilon & \nabla & \nabla & 10\;m & 10\;m & 10\;m & 10\;m & 0_{9\times 1} \end{array}\right]}^{2}\;$$

Initial measurement noise covariance:

(68) $${\hat{R}}_{0}=\mathrm{diag}{\left[\begin{array}{cc} 10\;m & 10\;m \end{array}\right]}^{2}.$$

**Figure 2:**
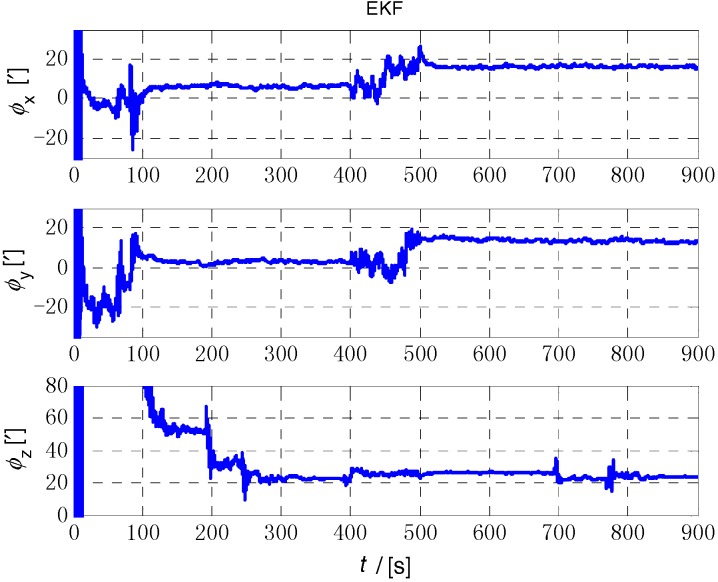
The initial alignment simulation results of EKF method. The three figures are the attitude error of *x* axis (pitch), *y* axis (roll) and *z* axis (yaw) respectively.

**Figure 3:**
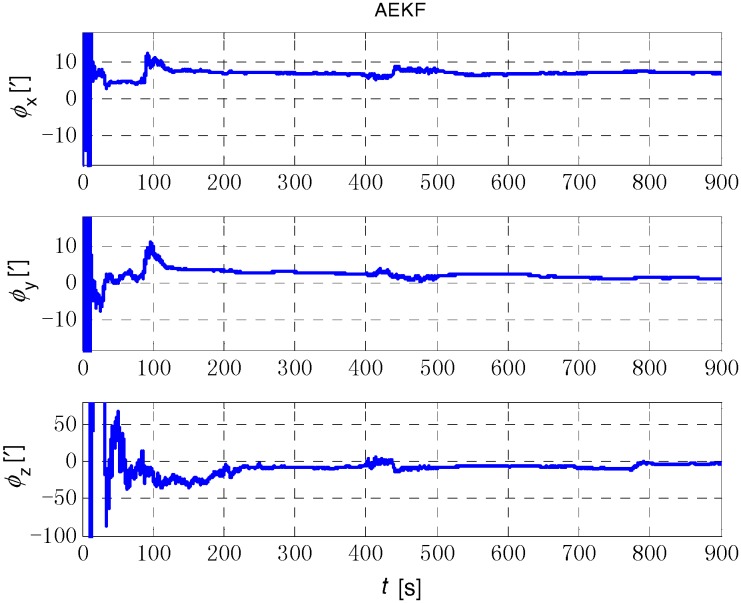
The initial alignment simulation results of AEKF method. The three figures are the attitude error of *x* axis (pitch), *y* axis (roll) and *z* axis (yaw) respectively.

**Figure 4:**
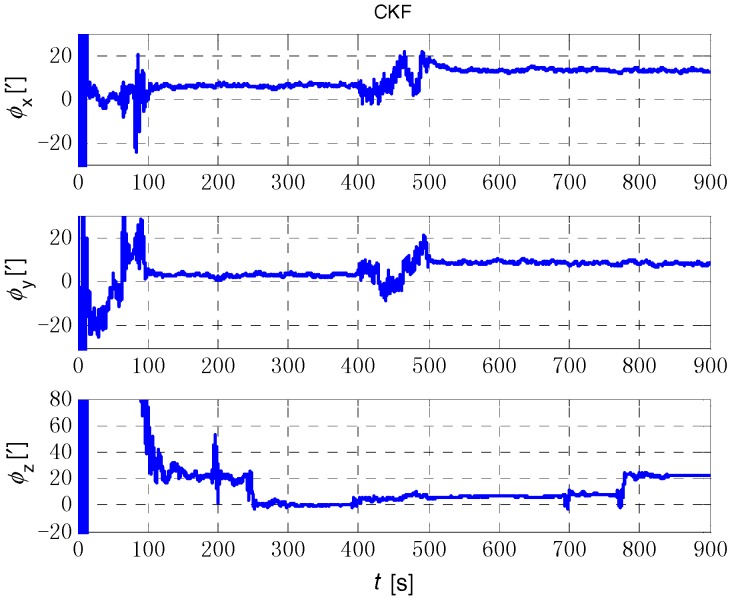
The initial alignment simulation results of CKF method. The three figures are the attitude error of *x* axis (pitch), *y* axis (roll) and *z* axis (yaw) respectively.

**Figure 5:**
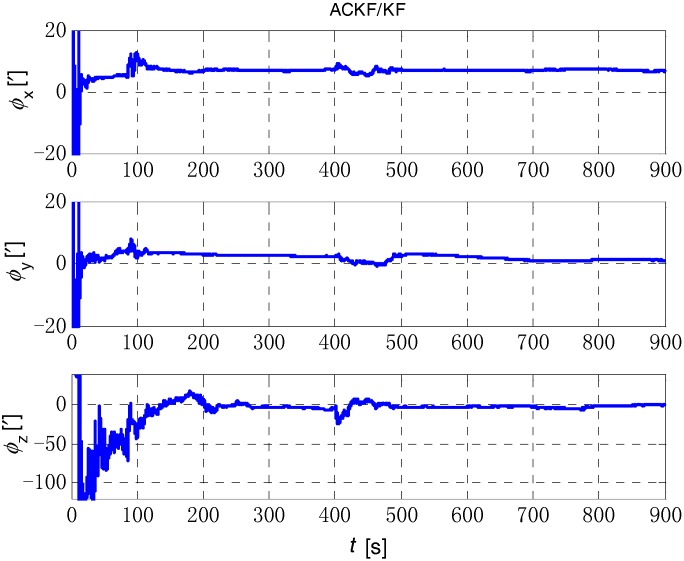
The initial alignment simulation results of ACKF/KF method. The three figures are the attitude error of *x* axis (pitch), *y* axis (roll) and *z* axis (yaw) respectively.

The initial alignment simulation was performed by EKF, AEKF, CKF and ACKF/KF respectively under the above simulation conditions, and the alignment results are shown in Figure 2, Figure 3,Figure 4 and Figure 5. Here, $\phi_{x}$, $\phi_{y}$ and $\phi_{z}$ mean the pitch, roll, and yaw respectively; the horizontal axis of the graph is time (s); the ordinate is the estimation error of attitude angle (′).

**Figure 6:**
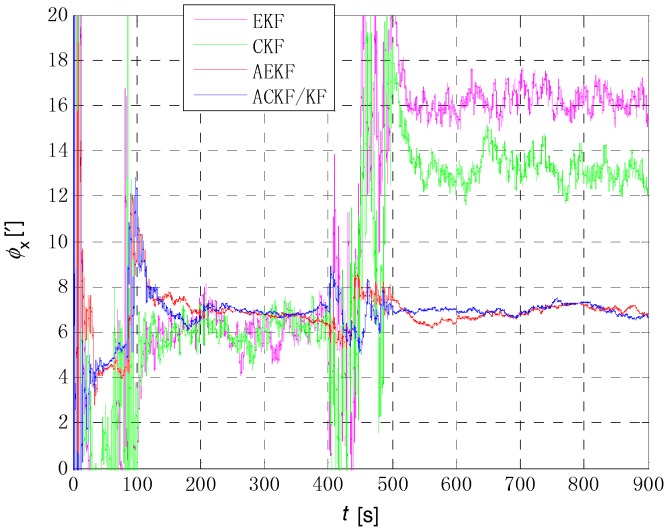
The comparison of estimation error of pitch angle. The pink line denotes the estimation by EKF, the green line denotes the estimation by CKF, the red line denotes the estimation by AEKF, and the blue line denotes the estimation by ACKF/KF.

**Figure 7:**
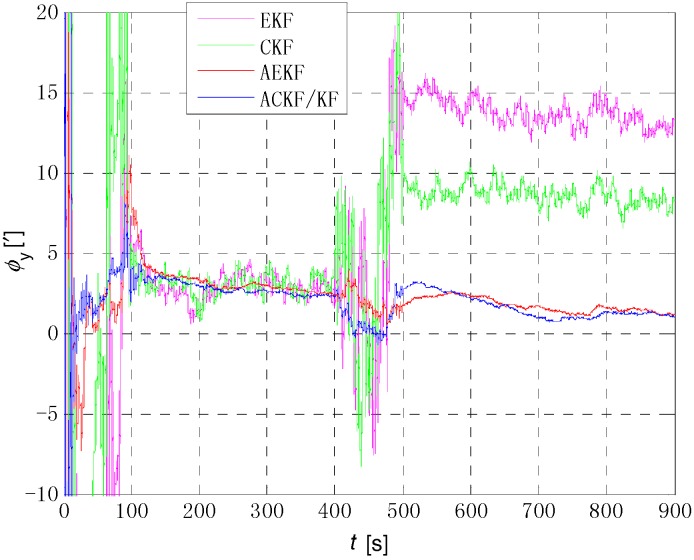
Comparison of estimation error of roll angle. The pink line denotes the estimation by EKF, the green line denotes the estimation by CKF, the red line denotes the estimation by AEKF, and the blue line denotes the estimation by ACKF/KF.

**Figure 8:**
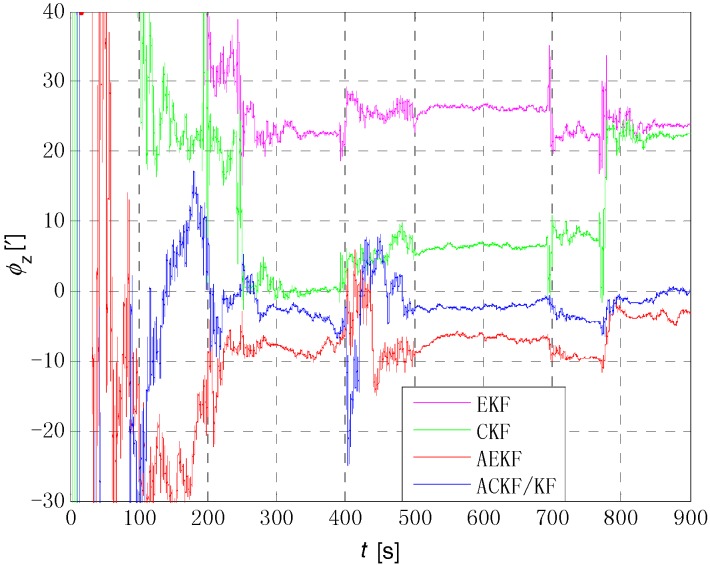
Comparison of estimation error of yaw angle. The pink line denotes the estimation by EKF, the green line denotes the estimation by CKF, the red line denotes the estimation by AEKF, and the blue line denotes the estimation by ACKF/KF.

Figure 6, Figure 7 and Figure 8 show the comparison of the four algorithms, in which the pink line denotes the estimation by EKF, the green line denotes the estimation by CKF, the red line denotes the estimation by AEKF, and the blue line denotes the estimation by ACKF/KF. It is obviously that the influence of measurement noise disturbance on the four algorithms is different. As the EKF and CKF algorithms still use the initial value of the measurement noise covariance during the increase of noise because of the lack of estimation of measurement noise, the filtering results are affected and the filtering accuracy is seriously reduced. Moreover, the estimation did not return to the original accuracy after the noise interference. As a result, the EKF and CKF cannot meet the accuracy requirements due to measurement noise interference during the alignment process. 

Due to the Sage-Husa adaptive filter, the filter accuracy and stability of ACKF/KF and AEKF are much better than those of EKF and CKF. However, the system of odometer aided SINS initial alignment with large misalignment angle is seriously nonlinear as shown in Section 2, the linearization truncation error in EKF somehow limits its estimation accuracy. The accuracy of the horizontal misalignment angle estimation of ACKF/KF and AEKF is approximately the same, but the accuracy of the ACKF/KF yaw misalignment angle is much higher. The mean and standard deviation of the estimation attitude error are listed in Table 1.

For the complex nonlinear model of the odometer aided SINS initial alignment with large misalignment angle, the EKF only uses the first order Jacobian matrix of the model to linearize, with large computational error. While the cubature points obtained in the CKF method according to the spherical-radial cubature rule can completely capture the mean and variance of Gaussian distribution variables, and the mean and variance can be accurate to the third-order or higher-order term of Taylor series expansion of nonlinear system. The Sage-Husa adaptive algorithm can estimate the noise in real time, thus improving the stability of the filtering results and making the convergence process more stable. The simulation results show that the proposed ACKF/KF algorithm can improve the accuracy and stability of the initial alignment.

### 4.2. Experiments and Analysis

**Figure 9:**
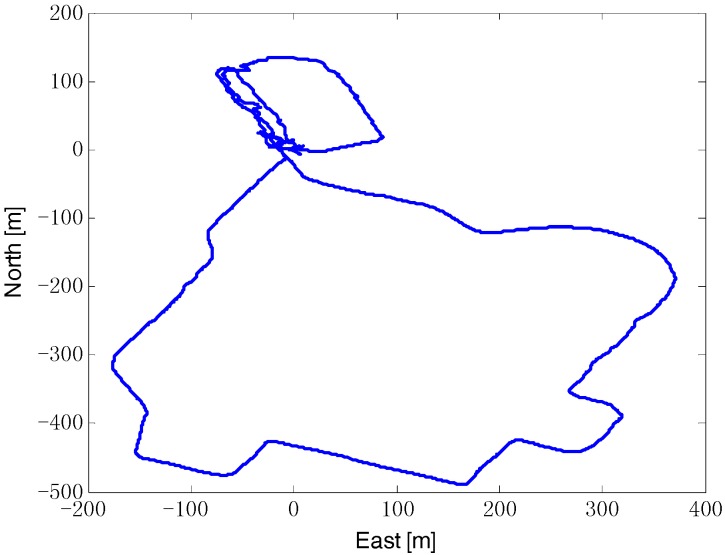
The vehicle test trajectory.

In order to verify actual performance of the proposed ACKF/KF algorithm, the initial alignment test was conducted by a vehicle equipped with SINS, odometer and GPS. The SINS is comprised of a triad of gyroscopes (drift 0.02°/h, noise 0.01°/(h/$\sqrt{\mathrm{Hz}}$) and accelerometers (bias 500 µg, noise 50 µg/$\sqrt{\mathrm{Hz}}$) at a sampling rate 100 Hz. The odometer scale factor error is about 0.2%, and the standard variance of measure noise is 0.02 m/s. The GPS is chosen as position reference, with the position accuracy less than 10 m and the velocity accuracy 0.01 m/s. And the attitude reference is given by SINS/GPS integrated navigation. The vehicle test trajectory is shown in Figure 9.

**Figure 10:**
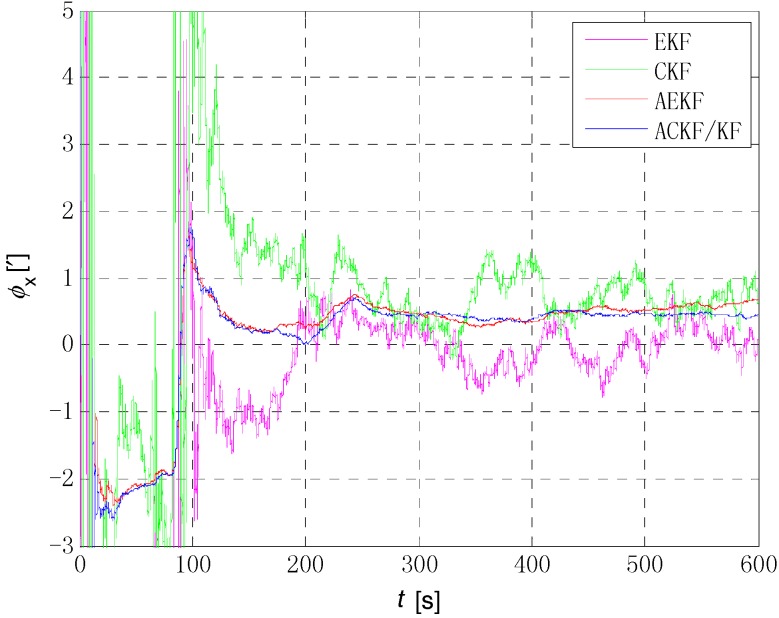
The estimated error of pitch angles. The pink line denotes the estimation by EKF, the green line denotes the estimation by CKF, the red line denotes the estimation by AEKF, and the blue line denotes the estimation by ACKF/KF.

**Figure 11:**
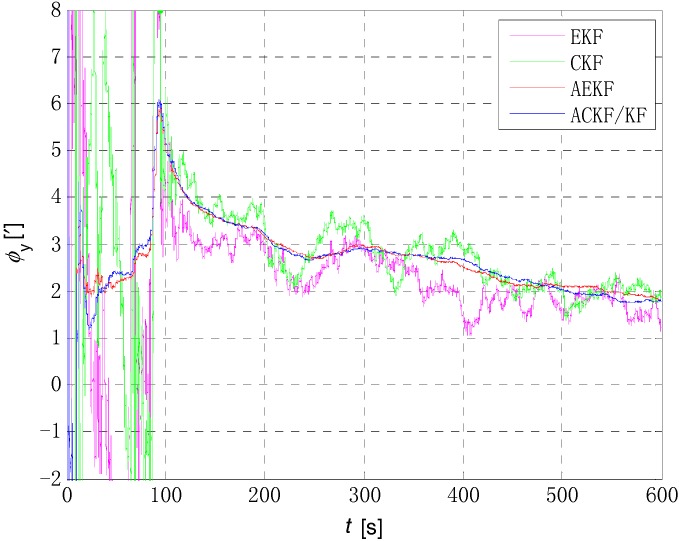
The estimated error of roll angles. The pink line denotes the estimation by EKF, the green line denotes the estimation by CKF, the red line denotes the estimation by AEKF, and the blue line denotes the estimation by ACKF/KF.

**Figure 12:**
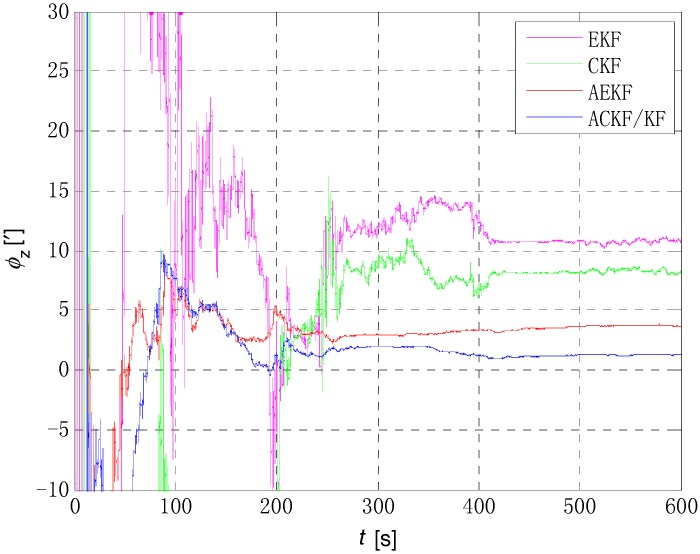
The estimated error of yaw angles. The pink line denotes the estimation by EKF, the green line denotes the estimation by CKF, the red line denotes the estimation by AEKF, and the blue line denotes the estimation by ACKF/KF.

The initial alignment error of pitch, roll and yaw are shown in Figure 10, Figure 11 and Figure 12 respectively, in which the pink, green, red and blue lines denote the estimation by EKF, CKF, AEKF and ACKF/KF respectively. It is obvious that the estimation process of ACKF/KF is much more smoothly than EKF and CKF, and the estimation precision is higher than AEKF. And the difference in Figure 12 is even more pronounced, in which the final estimated results of yaw is 10.7311′ of EKF, 8.2875′ of CKF, 3.7184′ of AEKF and 1.2627′ of ACKF/KF. The initial alignment results of attitude error in details are shown in Table 2. The initial alignment results are consistent with theoretical analysis and simulation results. Therefore, the proposed ACKF/KF method can accomplish the initial alignment with large misalignment angle well without a priori knowledge about the measurement noise, and the alignment speed and precision are significantly improved compared with EKF algorithm.

## 5. Conclusions

This paper has proposed an improved initial alignment method for land-vehicle SINS in-motion with large misalignment angle. There are two main points in this paper, one of them is the establishment of the linear observation equation of Kalman filter by deducing the odometer/ gyroscope dead reckoning error equation with large misalignment error, which sharply reduces the calculation of the initial alignment. The other is the improved ACKF/KF method, which combines the advantages of CKF and Sage-Husa adaptive filter. The simulation results show that the accuracy, stability and robustness is much better than the EKF, CKF and AEKF when the measurement noise changed during the initial alignment. Experiments show that the ACKF/KF method can accomplish the initial alignment with large misalignment angle in-motion.

## Figures and Tables

**Table 1:** Estimation of attitude error.

Algorithm	Mean	Standard Deviation
EKF	[16.2312′ 13.1301′ 23.7061′] ^T^	[0.4373′ 0.5588′ 0.6972′] ^T^
AEKF	[7.0033′ 1.3920′ −3.5554′] ^T^	[0.1118′ 0.1490′ 0.5211′] ^T^
CKF	[12.9795′ 8.1662′ 22.2739′] ^T^	[0.4318′ 0.5020′ 0.6329′] ^T^
ACKF/KF	[6.8852′ 1.2172′ −0.7645′] ^T^	[0.2279′ 0.0787′ 0.7646′] ^T^

**Table 2:** Initial alignment results of attitude error.

Algorithm	Mean	Standard Deviation
EKF	[0.139′ 1.7329′ 10.7311′] ^T^	[0.2296′ 0.2498′ 0.1677′] ^T^
AEKF	[0.6441′ 1.9502′ 8.2875′] ^T^	[0.1748′ 0.1984′ 0.1723′] ^T^
CKF	[0.5891′ 1.9932′ 3.7184′] ^T^	[0.0206′ 0.0920′ 0.0379′] ^T^
ACKF/KF	[0.4495′ 1.8672′ 1.2627′] ^T^	[0.0437′ 0.0853′ 0.0352′] ^T^
